# Chromatographic Comparison of Commercially Available Columns for Liquid Chromatography in Polar Pesticide Detection and Quantification Using a Score-Based Methodology

**DOI:** 10.3390/foods13193131

**Published:** 2024-09-30

**Authors:** Emanuela Verdini, Tommaso Pacini, Serenella Orsini, Stefano Sdogati, Ivan Pecorelli

**Affiliations:** Chemistry Department, Istituto Zooprofilattico Sperimentale dell’Umbria e delle Marche “Togo Rosati”, 06126 Perugia, Italy

**Keywords:** glyphosate and polar pesticides, chromatographic separation, columns comparison, mass spectrometry, score-based methodology

## Abstract

The detection and quantification of polar pesticides in liquid chromatography coupled with mass spectrometry present significant analytical challenges. This study compares the performance of three LC columns (Hypercarb™, Raptor Polar X™, and Anionic Polar Pesticide™) in separating and quantifying eleven polar pesticides in chicken eggs using a score-based methodology. Analytes include glyphosate, its metabolites, and other high-polarity pesticides like Ethephon, Glufosinate, and Fosetyl aluminum, included in the EU’s official control plan. Polar pesticides, characterized by high polarity and hydrophilicity, lead to analytical issues such as poor retention and unconventional peak shapes with traditional reversed-phase methods. Their weak interaction with hydrophobic stationary phases complicates separation, necessitating specific stationary phases to enhance retention and selectivity. This study evaluates these columns’ efficacy in complex matrices like chicken eggs and other food samples. Chromatographic separation was performed using a UPLC system coupled with a Q-TOF mass spectrometer; extraction and purification involved freeze-out, centrifugation, and filtration steps. The study highlights the critical role of column selection in achieving accurate and reliable separation and quantification of highly polar analytes in matrices of animal origin, offering in the meantime an easy-to-apply methodology of selection for the right determination of the best chromatographic column for different purposes.

## 1. Introduction

The accurate detection and quantification of polar pesticides in various matrices, particularly products of animal origin, remain a significant analytical challenge in the field of liquid chromatography coupled with mass spectrometry (LC-MS). The primary aim of this study is to conduct a comprehensive comparison of different liquid chromatographic (LC) columns designed specifically for polar pesticides, evaluating their efficacy in terms of separation and quantification.

Polar pesticides, due to their unique physicochemical properties, pose numerous challenges in separation and determination when analyzed using LC-MS techniques. Glyphosate is the most well-known pesticide belonging to this class; however, several other pesticides have also been approved and widely used due to their cost-effectiveness and efficiency [1]. The most common high-polarity pesticides (HPPs), showing increased usage due to their efficacy and the emergence of glyphosate-resistant weeds worldwide, include growth regulators (Etephon, Chlormequat), herbicides (Glufosinate, Paraquat, Diquat), and fungicides (Fosetyl Aluminum). Over time, the necessity to control polar pesticides has evolved, extending to their metabolites and byproducts, such as AMPA and N-Acetyl AMPA (glyphosate metabolites) or N-Acetyl Glufosinate and MPP (glufosinate metabolites).

The presence of these molecules in products of animal origin is a major concern for human health due to their easy transfer from agricultural products to animal tissues, along with their stability [2]. To monitor the presence of polar pesticides in food and ensure compliance with the EU maximum residue levels (MRLs), the European Commission promulgated a Regulation in 2022 providing for a multi-year control of pesticide residues in matrices of animal and plant origin, including ammonium glyphosate and glufosinate, and their metabolites, in bovine, pig and poultry fat, chicken eggs, bovine liver, and cow’s milk [3,4,5].

All these compounds exhibit high polarity and hydrophilicity, which often lead to several analytic issues, such as poor retention and unconventional peak shapes using the classic reversed-phase analytical methodology. The weak interaction of polar pesticides with the hydrophobic stationary phase results in inadequate separation and co-elution issues that complicate the analytical process [6,7].

Furthermore, the compatibility of these pesticides with various mobile phase compositions further complicates their chromatographic behavior, necessitating the use of specialized stationary phases that enhance retention and selectivity for polar analytes. The analysis of polar pesticides, especially in food and animal origin products, requires specific single-residue analytical methods due to their chemical characteristics, complicating all the steps of the analytical process, from extraction to chromatographic determination.

Several analytical techniques have been developed and validated over the years to detect and quantify polar pesticides and their metabolites in food of animal origin, such as ELISA [8,9], GC-MS/MS (with derivatization) [10], and LC-MS/MS [11,12,13,14,15,16].

Considering that most of these analytical methods have several drawbacks, particularly the limited range of analytes detected and quantified (most studies are limited to AMPA and glyphosate) and the necessity of derivatization to improve method performances, new analytical methods have been developed in recent years. The current trend focuses on the validation of new analytical methods compliant with SANTE requirements [17], which can lead to the simultaneous detection and quantification of a wide range of polar analytes with high selectivity, precision, and accuracy through an easy, reproducible, and efficient extraction and purification process (QuPPe) [18,19,20].

While extensive research has been conducted to identify the optimal reversed-phase columns for a broad range of polar molecules, the investigation into stationary phases with a high affinity for polar analytes is relatively scarce [21].

One of the first used chromatographic columns for polar pesticide separation is Hypercarb™ from Thermo, which was used by most of the applications since a few years ago. In recent years, manufacturers have engineered new stationary phases that have allowed to broaden the range of options for the separation of polar pesticides.

Previous studies have primarily focused on characterizing columns with embedded polar groups or hydrophilic end-cappings designed to improve the separation of basic and neutral compounds under high aqueous conditions [7]. However, these studies have not sufficiently addressed the specific needs and challenges associated with polar pesticide analysis, particularly in complex matrices such as products of animal origin. Notably, columns such as Hypercarb™ with porous graphitic carbon [18,19,20,21,22], Reversed-phase C18 columns [22,23,24,25], Normal-Phase Silica columns [26], Negative charge/hydrophobic linkages columns or Positive charged/hydrophilic linkages columns [27,28,29,30,31], Capillary GC columns [32], and Hydroxide-selective anion-exchange columns [33,34] have been used and compared in the past, each showing different advantages and disadvantages [21].

This type of investigation, evolving through the comparison of different columns with varying characteristics, mainly for fruit and vegetable matrices, has created a significant gap in the literature. This underscores the need for targeted research to evaluate and compare the performance of LC columns specifically tailored for polar pesticides in order to reliably detect and quantify a wide range of these analytes in food of animal origin. No published research to date has systematically compared LC columns for the analysis of polar pesticides in animal-derived products, representing a critical area for food safety and toxicological studies [7,35].

**Figure 1:**
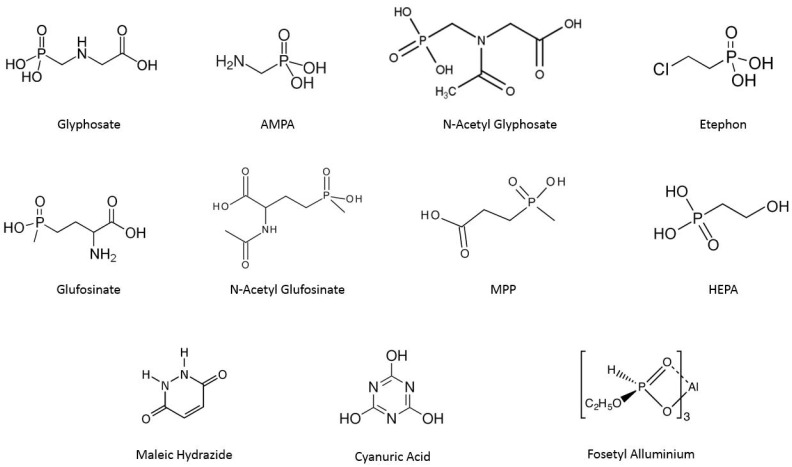
Chemical Structures of main polar pesticides; AMPA, N-Acetyl Glyphosate are metabolites of Glyphosate, N-Acetyl Glufosinate G and MPP are Glufosinate metabolites.

This study aims to fill this research gap by thoroughly examining the characteristics and performance of three different chromatographic columns compatible with polar pesticide separation through the detection of eleven different analytes, reported in Figure 1, in an animal origin matrix (chicken egg). By identifying the most suitable column for this purpose, this study will provide valuable insights for scientists working in toxicology and related fields, enhancing the accuracy and reliability of polar pesticide detection in products of animal origin.

## 2. Materials and Methods

### 2.1. Reagents, Standard Solution, and Materials

Reference standard solutions of AMPA, Ethephon, Glufosinate, Glyphosate, HEPA, Maleic Hydrazide, MPP, N-acetyl glyphosate in water/acetonitrile (9:1 *v*/*v*) (1000 μg/mL); Fosetyl aluminum, N-acetyl glufosinate in water/acetonitrile (9:1 *v*/*v*) (100 μg/mL); and reference materials of Cyanuric acid as a pure solid (purity 99.3%) were purchased from Lab Instruments Srl (Castellana Grotte, Italy). Ethylenediaminetetra acetic acid disodium salt dihydrate (EDTA) was obtained from Merck (Darmstadt, Germany) and Polygoprep™ 300-30 C18 from Macherey-Nagel GmbH & Co. KG (Düren, Germany). Methanol (MeOH) and Acetonitrile (ACN) were obtained from Carlo Erba Reagents Srl (Milan, Italy). All solvents used were of LC–MS or analytical grade. Unless otherwise specified, water purified by a Milli-Q system (Millipore, Merck KgaA, Darmstadt, Germany) was used for sample preparation and analysis.

### 2.2. Samples

Matrix-matched standards of chicken eggs were prepared by spiking blank samples at 0.005 mg/kg for glyphosate, AMPA, Ethephon, HEPA, N-acetyl glufosinate, Fosetyl Al, Cyanuric Acid, Maleic Hydrazide, and 0.002 mg/kg for Glufosinate, MPP, and N-acetyl glyphosate, representing the central level of the calibration curve for the quantification of polar pesticides. All samples were ground using a knife mill (GRINDOMIX GM 300, Restek, Haan, Germany) with dry ice. The samples were stored at −20 °C until analysis.

### 2.3. Column Selection

The columns selected for this comparative study were chosen based on the characteristics of the analytes, i.e., high polarity and the subsequent lack of suitable stationary phases for their chromatographic separation. The selection focused on the capability of each column to show good separation for the selected analytes (Figure 1) without any restriction or preconception regarding the nature of the stationary phase. Ionic chromatography was excluded from the very beginning. The project began with a request initiated by our lab, inviting various column suppliers to participate. Suppliers interested in this project were asked to provide columns for the study along with their optimized chromatographic methods for detecting polar pesticides in animal-origin products. The columns supplied for this comparison were Raptor Polar X™ (RPX) from Restek, Via G. Miglioli 2A. Cernusco sul Naviglio (MI), Italy, and Anionic Polar Pesticide™ (APP) from Waters, Viale dell’ Innovazione 3, Milano, Italy.

The comparison was performed, including Hypercarb™ (HYC) from Thermo, Via San Bovio 3, Segrate (MI), Italy, already used in the validation of a new polar analytical method internally developed [36].

The characteristics of the selected columns are reported in Table 1.

Regarding the retention mechanism of these three columns, the PGC-Hypercarb is characterized by layers of hexagonally arranged carbon atoms linked by the same conjugated bonds, which are present in any large polynuclear aromatic hydrocarbon. The retention mechanism of this peculiar stationary phase for polar analytes is characterized by the so-called “polar retention effect of graphite”: as the polarity of the analyte increases, despite what could be expected, the retention time increases due to a particular orbital overlap between the conductivity electrons in graphite and lone pair and π electrons in analytes which locally polarize the stationary phase [37,38].

The hybrid phase, which characterizes the Raptor Polar column, is constituted by a single ligand capable of retaining polar analytes through a balance of two retention mechanisms: hydrophilic interaction chromatography (HILIC) and ion-exchange approach. It is possible to promote one mechanism or the other by simple changes in mobile phase conditions, leading to the possibility of retaining and separating a wide number of polar molecules with different mechanisms in the same analysis. In detail, as the percentage of water content increases, the ion exchange mechanism takes over as the dominant mode of retention [39].

The anionic polar pesticide, finally, is characterized by a diethylamine stationary phase acting with a classic hydrophilic interaction mechanism.

### 2.4. Analytical Method

#### 2.4.1. Extraction Method

The analytical procedure was reported elsewhere and is briefly summarized below: [36].

Two grams of homogenized chicken eggs are extracted with 8 mL of MilliQ Water and 10 mL of MeOH with 1% formic acid. The freeze-out was accomplished by freezing the treated samples at −80 °C for 15 min and then centrifuging at 4 °C in an ultracentrifuge (15,000 R.C.F.). A total of 2 mL of supernatant was purified in a centrifuge tube by 100 mg of C18 sorbent and 2 mL of acetonitrile. The samples were mixed, centrifuged at 0 °C for 10 min (12,000 R.C.F.), and filtered in plastic vials. The standards were added at the desired concentrations before injection (see Section 2.2). The acceptability criteria for the detection of the standards were set in compliance with the SANTE document, emphasizing the importance of matching retention time and mass accuracy of the detected peak with a signal-to-noise ratio (S/N) ≥ 3.

The extraction methods considered at the beginning of the present study were the Quick Method for the Analysis of Numerous Highly Polar Pesticides in Foods Involving Extraction with Acidified Methanol and LC–HRMS Measurement in Foods of Animal Origin (QuPPe AO) [39] and the procedure developed by Herrera et al. [30].

The implementation made to the QuPPe protocol [39], in detail, referred to the improvement of the speed and time of centrifugation, the introduction of additional purification steps, and the modification of dilution factors in order to obtain the best compromise between sensitivity and the matrix effect and to improve LOQ for the selected analytes. Finally, a 20-fold dilution factor was chosen in the implemented method to reduce the matrix effect [36]. On the other hand, the protocol developed by Herrera et al. [30] is characterized by a high dilution factor, which was not compatible with the sensitivity of the equipment used in the study (HRMS).

The extraction method described above was part of an interlaboratory study performed in 2022–2023 involving five European laboratories, which underlined the efficiency of this protocol for polar pesticide quantification [40].

#### 2.4.2. Chromatographic Methods

The optimal chromatographic parameters used for polar analytes separation for both APPC and Raptor Polar X columns were not further investigated due to the prior optimization already performed and furnished by the suppliers. On the other hand, for the Hypercarb column, the optimization of parameters was determined in a previous study, which has already been published [36].

The characteristics of the different chromatographic methods are summarized below (Table 2, Table 3 and Table 4).

*Hypercarb*

The mobile phase for chromatographic separation was LC-MS grade Water containing 0.1% formic acid (MF A) and an LC-MS grade acetonitrile solution containing 0.1% formic acid (MF B), with the following gradient:

**Table 2:** Analytical Gradient for Hypercarb.

Time (min)	Flow Rate (mL/min)	% A	% B
0	0.5	35	65
5	0.5	90	10
11.5	0.5	90	10
11.51	0.5	35	65
13	0.5	35	65

The injection volume was 10 μL.

*Raptor Polar X*

The mobile phase for chromatographic separation was LC-MS grade Water containing 0.5% formic acid (MF A) and an LC-MS grade acetonitrile solution containing 0.5% formic acid (MF B), with the following gradient:

**Table 3:** Analytical Gradient for Raptor Polar X.

Time (min)	Flow Rate (mL/min)	% A	% B
0	0.5	35	65
5	0.5	90	10
11.5	0.5	90	10
11.51	0.5	35	65
13	0.5	35	65

The injection volume was 10 μL.

*Anionic Polar Pesticide*

The mobile phase for chromatographic separation was LC-MS grade Water containing 0.9% formic acid (MF A) and an LC-MS grade acetonitrile solution containing 0.5% formic acid (MF B), with the following gradient:

**Table 4:** Analytical Gradient for Anionic Polar Pesticide.

Time (min)	Flow Rate (mL/min)	% A	% B
0	0.5	10	90
4	0.5	85	15
13	0.5	85	15
18.5	0.5	10	90

The injection volume was 10 μL.

#### 2.4.3. Acquisition Method

The detection of the eleven analytes was performed using a UPLC system (Exion LC, AB Sciex) coupled with tandem mass spectrometry equipped with a Q-TOF 6600+ (AB Sciex) with ESI interface in negative acquisition mode, using a routine in-house validated method [36].

The mass spectrometry parameters were optimized for each analyte to maximize the sensitivity and selectivity of detection, including capillary voltage, collision energy, and ion source temperature (Table 5).

### 2.5. Column Selection Criteria

Once the comparative trials are completed, the column performances are evaluated using a total score-based methodology developed internally. Each column was assigned a Column Performance Score (CPS) based on its compliance with seven different set requirements. This method provides a new, effective way to compare different columns, which is easily extensible to similar cases. It addresses a gap in the literature that has so far been filled only by different statistical models [6,7,41].

The present method could simplify the comparison of columns by providing a straightforward, easily extensible scoring system.

#### 2.5.1. Conditioning or Passivation (Y/N)

Before the column is used, in many cases, depending on the stationary phase nature, a certain time of conditioning with mobile phases before the analysis is required. This fact is due to the necessity of activation of some active sites responsible for the correct retention of the analytes. This evaluation was performed based on the information obtained from the literature. The time wasted during the conditioning or passivation of the stationary phase, as well as the poor performances of the column itself when passivation is not applied, has been considered disadvantageous, increasing de facto the time of the analysis: for this reason, a score of 5 points was defined for analytical columns without particular conditioning necessities. Otherwise, 0 points were awarded.

#### 2.5.2. Peak Shape and Symmetry

Peak shape and peak symmetry are indeed key parameters for the evaluation of chromatographic column performance. In this case, these two parameters were considered when analyzing the characteristics of the chromatographic peaks: in the first instance, for evaluating the peak shape, the difference between single or double peaks was observed; a single one is a peak with one base, and only one apex, a double peak has one base but two separate apexes. A second time, for the evaluation of the peak symmetry, the distance from the peak centerline to the back slope divided by the distance from the peak centerline to the front slope, with all measurements taken at 10% of the maximum peak height, has been considered. Peaks characterized by a symmetry value (SV) between 0.7 and 1.5 were considered as symmetric, whereas peaks with SV out of this range were considered asymmetric. A score of 4 points was awarded for any single symmetric peak, 3 points for any single asymmetric peak, 2 points for any double symmetric peak, and 1 point for any double asymmetric peak. For AMPA and Glyphosate, the points were doubled due to the issues reported in the determination of these two molecules [36].

#### 2.5.3. Stability Test

The stability of the signal and the retention time were investigated as follows. Fifty replicates of the same sample, a mix of Glyphosate, N-acetyl Glyphosate, and Glufosinate ammonium in the matrix, have been analyzed. The analysis was performed using three different columns that exploited the optimized parameters supplied by the producer. For any analysis, the relative standard deviation (RSD) obtained for the area and retention time has been calculated. For the molecules with a RSD less or equal to 10%, 4 points were awarded, 3 points for RSD between 10 and 20%, 2 points for RSD between 20 and 30%, and 0 points for RSD > 30%.

#### 2.5.4. Sensitivity Test

For sensitivity tests, analyses following document EUR 28,099 EN have been performed [42]. Ten samples were analyzed after the addition of a mixture of target analytes at a concentration level close to the estimated LOD, and a 5-point calibration line was registered (extraction and purification were used for the optimized method). The LOD of each compound was determined from the calibration lines and the standard deviation of the signals. The data for each analyte were obtained by comparing the values calculated with the three columns, and the highest score (5) was assigned to the one with the lowest LOD value and to the others in proportion (4 and 3 respectively for the one with the intermediate value and the one with the highest LOD value).

#### 2.5.5. Retention Factor (k)

Considering the same analysis performed in Section 2.5.2, the k factor for each analyte was calculated in any chromatographic system; the *k* factor is calculated as:

$$k=\frac{t_{r}-t_{0}}{t_{0}}$$

where $t_{r}$ is the retention time of each analyte and $t_{0}$ is the hold-up time. For any specific column, 1 point was assigned for any peak with a k factor value between 1 and 10; for peaks with a k value > 10, 0 points were awarded.

#### 2.5.6. Chromatographic Column Life

Around 500 replicates from the same sample, spiked with polar pesticides in the matrix, have been analyzed. For any analyte detected, the RSD for the retention time and the RSD for the peak symmetry have been calculated. The injection sequence was the following:

- 200 initial injections;
- 75 injections for stability and sensitivity tests;
- 200 final injections.

After the comparison of the RSD obtained for each column for peak symmetry and retention time of the target analytes, a score of 4 points was assigned to the column with the lower RSD for both parameters, 3 points for the second column, and 2 points for the last one.

#### 2.5.7. Supplementary Extra Molecules

An extra score of 0.5 points for the correct separation and characterization of each additional analyte not provided by the validated method but suggested by the supplier, and one point for any molecule included in the present study.

At the end of the experimental phase, the sum of the seven scores was used to compare the three columns and identify the one that best fits our scopes.

## 3. Results and Discussion

The chromatographic performances of the three selected columns have been evaluated based on the parameters reported above. The analysis revealed significant differences in the chromatographic behavior of the polar pesticides across the different stationary phases. The Hypercarb™ column demonstrated superior retention and peak shape for glyphosate and its metabolites, while the Raptor Polar X™ and Anionic Polar Pesticide™ columns exhibited better performance for other analytes such as Fosetyl aluminum and Ethephon.

The separation efficiency, evaluated through the resolution between peaks, was generally higher for the Hypercarb™ column, likely due to the unique properties of porous graphitic carbon that enhance interactions with highly polar compounds. However, the Anionic Polar Pesticide™ column provided a good balance between retention and peak shape for a broader range of analytes, making it a versatile option for multi-residue analysis.

### 3.1. Conditioning or Passivation Results

Among the columns considered in the present study, nevertheless, despite the wide differences among their stationary phases, the only one that did not need conditioning or passivation before the analysis was the APP. Before use, Hypercarb columns and pre-columns have to be thoroughly primed to cover certain active sites on the surface, with solutions containing planar molecules such as chlorophyll and anthocyanins in order to accelerate the priming period. This step has been performed through multiple injections of a QuPPe extract of spinach, prepared by dissolving 100 mg of spinach extract in 20 mL methanol + 1% FA-H_2_O 1:1. A total of 10–15 injections of spinach extract are typically required for the pre-column and ca. 50 injections were used for the column and pre-column combined (50 mL of solution each time) [43].

In the same way, Raptor polar X needs to passivate the system with a methanolic solution of Methylenediphosphic acid (Mendronic Acid) before use. The passivating solution had to be injected several times through the injector, excluding the column that had already been passivated prior to shipping. The flow is directed to the waste, and the mobile phase flow rate has been set at 0.4 mL/min, with 10 full loop injections and allowing the mobile phase to flow for 1 min [44].

The summary of the passivation test is reported in Table 6.

### 3.2. Peak Shape and Symmetry Results

In order to perform this evaluation, as already explained in Section 2.5.2, two different indicators have been taken into consideration for peak shape and symmetry, such as single or double apex corresponding to a single base and ratio of the distances from the frontline and backline of the peak from the centerline. We have considered five different possibilities with different scores: UASP = Unique Apex Symmetric Peak; UAAP = Unique Apex Asymmetric Peak; DASP = Double Apex Symmetric Peak; DAAP = Double Apex Asymmetric Peak; NA = No Apex (absent peak or indistinguishable from noise). Table 7 shows the results for this criterion.

**Figure 2:**
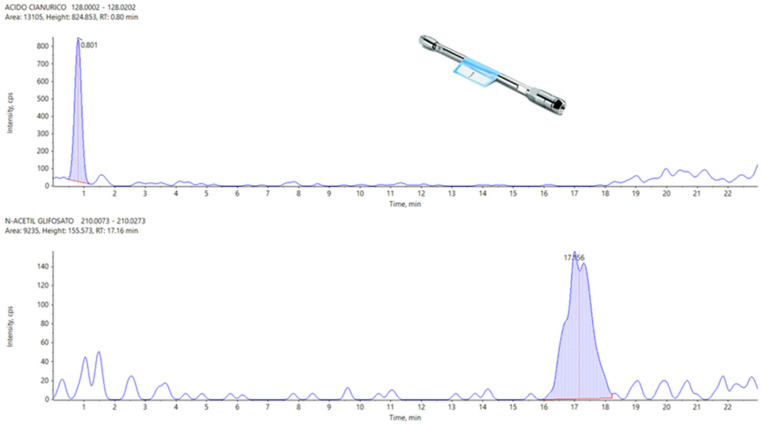
Cyanuric acid (**top**) and N-Acetyl Glyphosate (**bottom**) peaks, the concentration of which is 0.005 mg/Kg, with an APP chromatographic column.

For Peak shape and Peak symmetry combined criteria, APP seems to be the best column, followed by the Hypercarb and then the Raptor polar. N-Acetyl Glufosinate and Cyanuric Acid were not detected with Raptor Polar X, but they are separated and detected on APP, Figure 2 as reference, and Hypercarb.

### 3.3. Stability Test Results

After the fifty replicates of the samples spiked with Glyphosate, Glufosinate Ammonium, and N-Acetyl Glufosinate, the RSD for the Area and the retention time of the peaks have been calculated, and the results are listed in Table 8.

The analysis of this criterion has shown better performances for Hypercarb and Raptor polar than for APP, which received 17 points against 21 and 20, respectively. This result was particularly affected by the results obtained for the APP area standard deviation of the glyphosate peak; on the contrary, the Hypercarb has shown great stability for both retention time and area for this challenging analyte.

**Figure 3:**
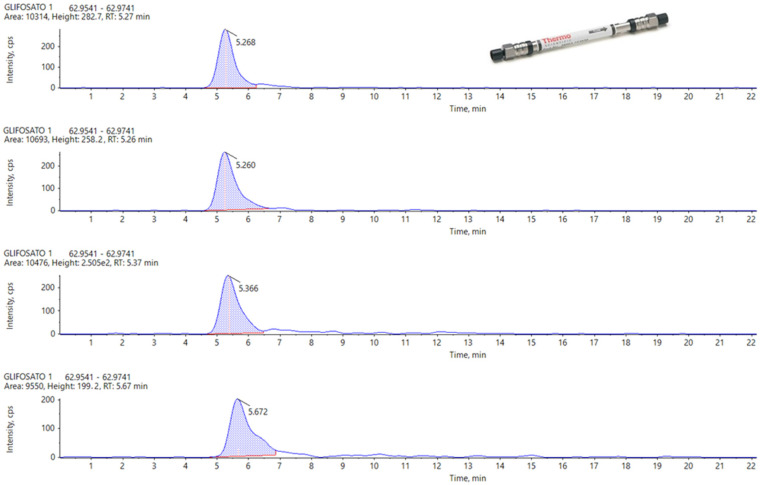
From Top to the Bottom: Glyphosate peak on Hypercarb, Injection Number 1, 15, 35 and 50.

**Figure 4:**
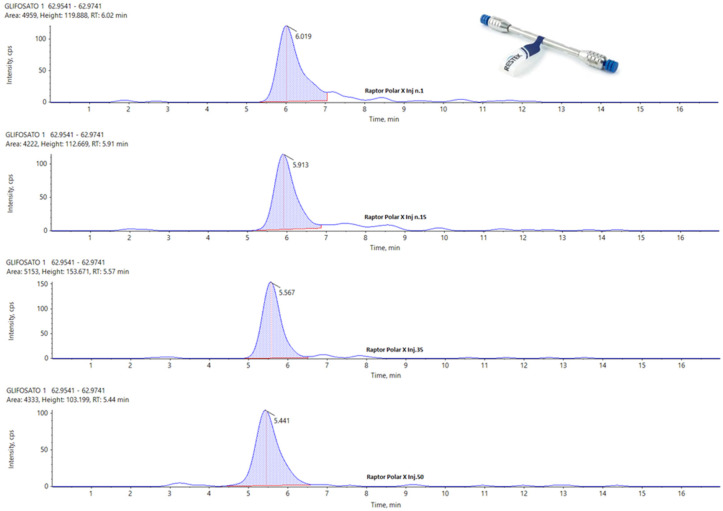
From Top to the Bottom: Glyphosate Peak on Raptor Polar X, Injection Number 1, 15, 35, and 50.

**Figure 5:**
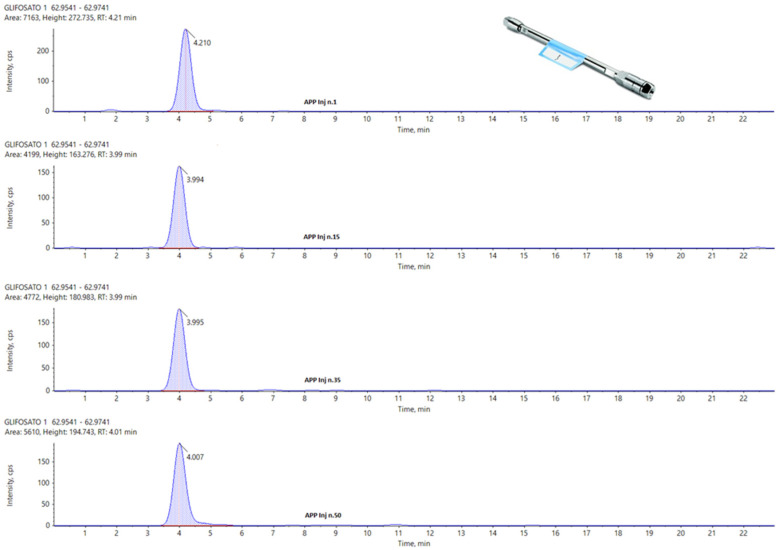
From Top to the Bottom: Glyphosate peak on APP, Injection Number 1, 15, 35 and 50.

In the following figures, a comparison among four chromatograms in different acquisition have been reported for glyphosate peak: the area and the retention time are much more reproducible for Hypercarb and Raptor polar (Figure 3 and Figure 4) than for APP (Figure 5).

Figure 3 and Figure 4 illustrate that, although the chromatographic peak of Glyphosate appears less resolved than the same analyte in APP, it exhibits a more stable peak area compared to APP. In APP, the peak area ranges from a minimum of 4199 to a maximum of 7163, almost doubling. This significant fluctuation in APP suggests potential issues with consistency and precision in quantifying Glyphosate.

During the repetitions, the variations observed in the peak area for the other two columns are much more controlled, showing no significant fluctuations. This consistency is particularly evident in the Hypercarb column, which demonstrates the best results in terms of the repeatability of the Glyphosate area. The consistent peak areas with the Hypercarb column highlight its reliability and suitability for precise Glyphosate analysis.

Conversely, the RT (retention time) variations are minimal across all columns, with no significant differences among the three columns for the three analytes considered. This suggests that while peak resolution and area stability might vary, the retention times are consistent, indicating robust method performance across different columns. These findings underscore the importance of selecting appropriate columns for specific analytes to ensure accurate and reliable analytical results.

### 3.4. Sensitivity Test Results

The results of the sensitivity test, as described in Section 2.5.4, are listed in Table 9.

The Hypercarb seems to be the most sensitive column, with a result similar to that of the APP. The Raptor Polar X partially failed this test, in particular for what concerns three analytes, N-Acetyl Glyphosate, Cyanuric Acid, and Maleic Hydrazide, which present peaks not detectable and confused with the background noise.

**Figure 6:**
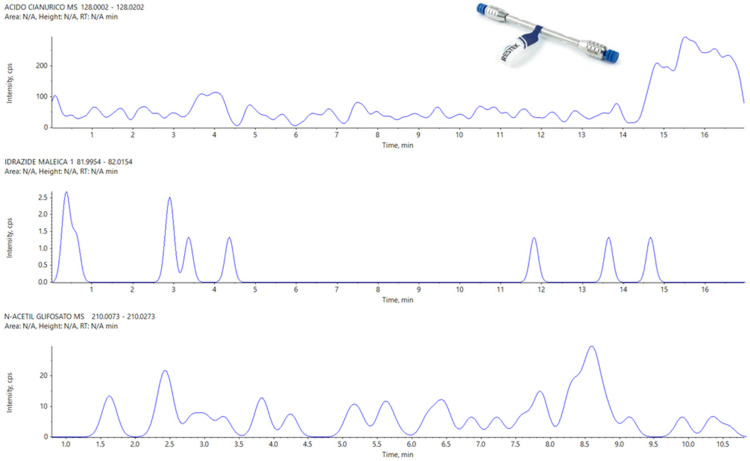
From Top to Bottom: Cyanuric Acid, Maleic Hydrazide, and N-Acetyl Glyphosate on Raptor Polar X at LOD.

**Figure 7:**
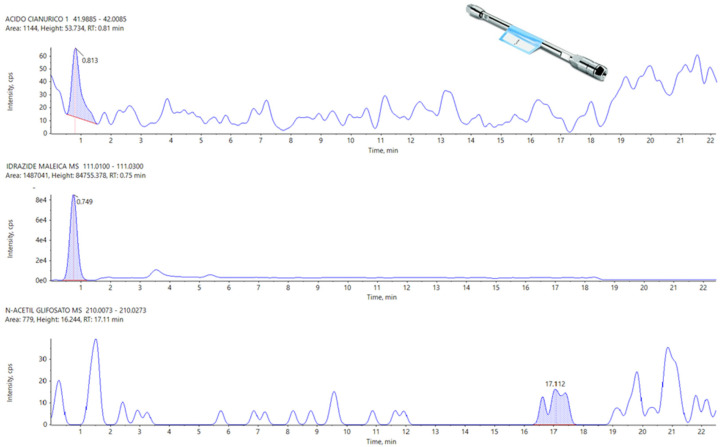
From top to bottom, Cyanuric Acid, Maleic Hydrazide, and N-Acetyl Glyphosate on the APP at LOD.

As observed at higher concentrations (see Section 3.2), the Raptor polar X is not efficient in the separation of Cyanuric acid and N-Acetyl Glyphosate. In many cases, we have observed even the absence of a peak to refer unequivocally to Maleic Hydrazide, so, even for this analyte, it was impossible to define a correct LOD with the Restek column (Figure 6. The peaks for these three analytes (Cyanuric acid, Maleic Hydrazide, and N-Acetyl Glyphosate) are reported in Figure 7 with APP for comparison.

### 3.5. Retention Factor (k) Results

For all the considered analytes and the three columns investigated, retention time, k values, and relative scores are reported in Table 10:

Considering these values, Hypercarb and APP gain the same points, 7 out of 11. For what concerns the Raptor Polar X, almost every k value is in a range > 10 or <1, and for this reason, this column gains 1 point for Glyphosate only.

### 3.6. Chromatographic Column Life Results

In order to evaluate the chromatographic column life, careful observation of the reproducibility of peak symmetry and retention time for any considered analyte was necessary. The results of the calculation of RSD for RT and symmetry of each analyte’s peak after around 500 replicates, as described in Section 2.5.6, are reported in Table 11.

**Figure 8:**
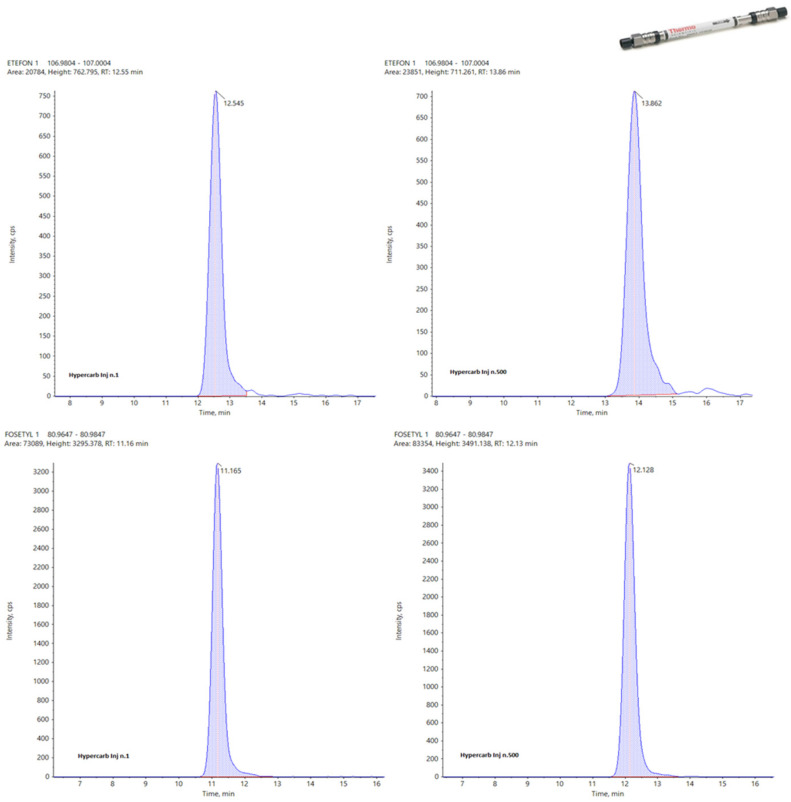
Peaks of Etephon (**top**) and Fosetyl Al (**bottom**) on Hypercarb at the first (**left**) and 500th injection (**right**).

**Figure 9:**
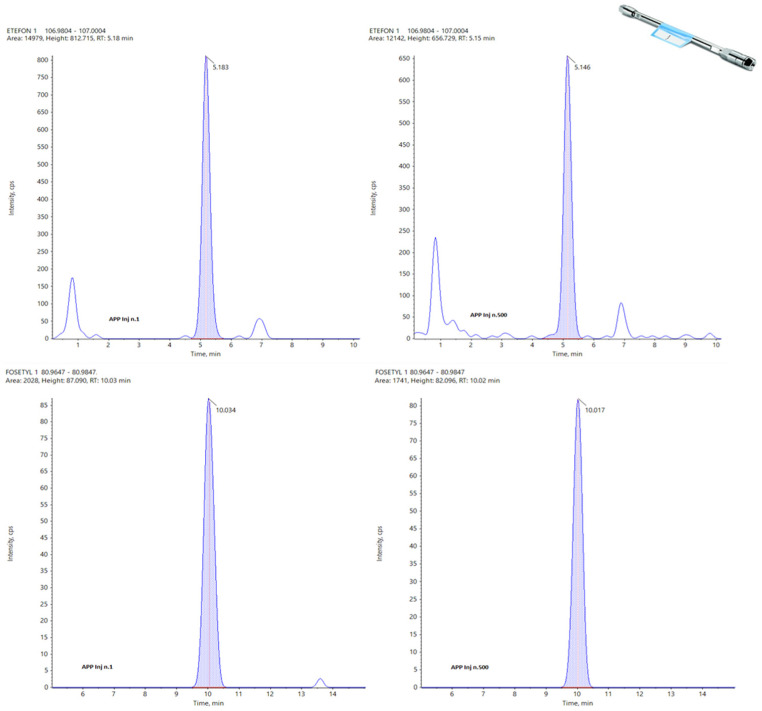
Peaks of Etephon (**top**) and Fosetyl Al (**bottom**) on APP at the first (**left**) and 500th injection (**right**).

**Figure 10:**
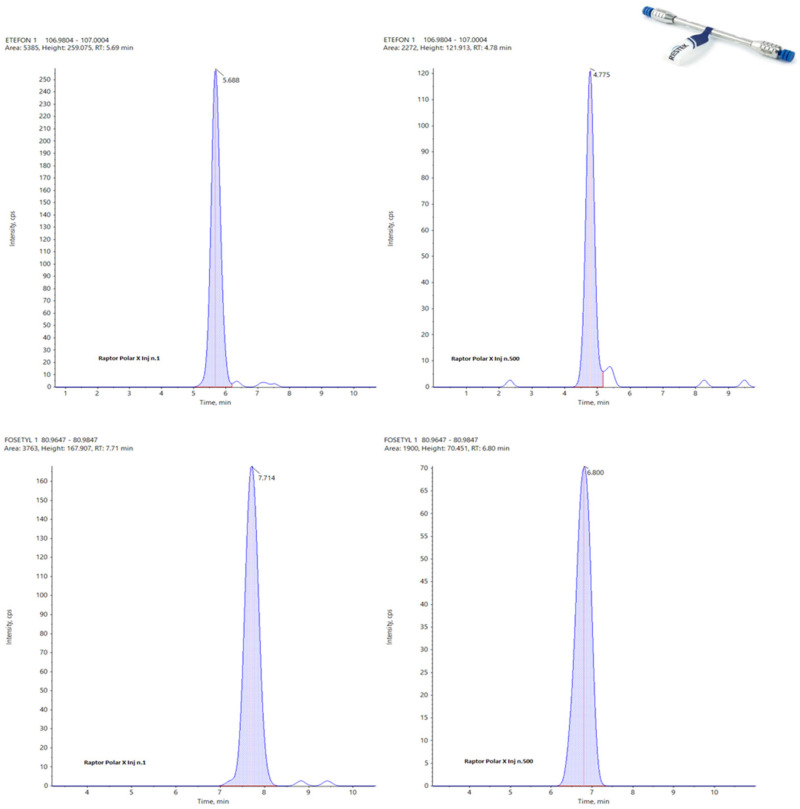
Peaks of Etephon (**top**) and Fosetyl Al (**bottom**) on Raptor Polar X at the first (**left**) and 500th injection (**right**).

Analyzing the data obtained for these three columns concerning their working life, it is easy to identify the APP largely as the best one with a total of 82 points against 64 awarded to the Hypercarb and only 44 for the Raptor Polar X. This last data is clearly affected by the impossibility for this column to detect N-Acetyl Glyphosate and Cyanuric acid, but, in general, the RSDs calculated for the APP are always very low with respect to the others. In the following figures, the same peak for two different analytes (Etephon and Fosetyl Al) at the first injection (left) and at the last one (right) are reported for Hypercarb (Figure 8), APP (Figure 9) and Raptor polar X (Figure 10).

### 3.7. Supplementary Molecules

The last criterion used is referred to the points for any correct separation of the analytes included in the validated analytical method (Table 12). In addition to that, results for extra-molecules correctly separated by the single columns are described in Table 13.

Even for this last criterion, the best columns seem to be APP, together with Hypercarb, with the same total score (14); on the contrary, Raptor polar X shows a quite low score due to a lower number of analytes separated and detected.

### 3.8. Methodological Improvement of Column Performance Evaluation in Comparison with QuPPe

The analytical performances of the chromatographic columns for APP and Hypercarb have already been shown in the QuPPe AO document through some exemplary LC-MS/MS chromatograms without reporting other objective parameters for their evaluation.

The QuPPe does not consider the use of the Raptor Polar X column for animal-origin products but only for Food of Plant Origin (QuPPe-PO-Method M1.9) [43].

The main differences concerning the chromatographic behavior of the other two columns considered in this study, compared to QuPPe AO, are highlighted below.

− Hypercarb, Comparison present study vs. QuPPe LC-MS/MS method M1.3:

The method proposed by QuPPe has been applied to chicken eggs. However, QuPPe’s study focuses on only eight analytes. Our study expands on this by including additional analytes compared to method M 1.3, such as AMPA, cyanuric acid, and N-acetyl glyphosate. Moreover, QuPPe reports concentrations of these analytes in chicken egg extracts at 0.05 mg/kg, while our study evaluated peak shape and RSD at much lower concentrations: 0.005 mg/kg for glyphosate, AMPA, ethephon, HEPA, N-acetyl glufosinate, fosetyl-Al, cyanuric acid, and maleic hydrazide, and 0.002 mg/kg for glufosinate, MPP, and N-acetyl glyphosate. Lastly, while the QuPPe document presents analyte chromatograms with subjective information on peak shape, our work provides an objective evaluation based on parameters such as peak shape, stability, peak symmetry, column life, and sensitivity.

− APPC, Comparison present study vs. QuPPe LC-MS/MS method M1.6:

The method proposed by QuPPe does detect the same analytes quantified in our study using APPC; moreover, it has not been applied to chicken eggs. As shown in Figure 6 (solvent: MeOH), Figure 7 (bovine liver), Figure 8 (milk), and Figure 9 (butter oil) of QuPPe, the method was tested on different matrices. Even in this case, the QuPPe study presents analyte chromatograms with subjective information on peak shape alone.

## 4. Conclusions

This study represents a pioneering effort in the literature by comparing different columns for the detection of polar pesticides in products of animal origin. Our analysis has shown that the Anionic Polar Pesticide column is the most effective for detecting polar pesticides in animal-derived products, particularly in chicken eggs. This column achieved a total score of 207 points, outperforming the Hypercarb and Raptor Polar X columns, which scored 193 and 142 points, respectively (Table 14).

**Figure 11:**
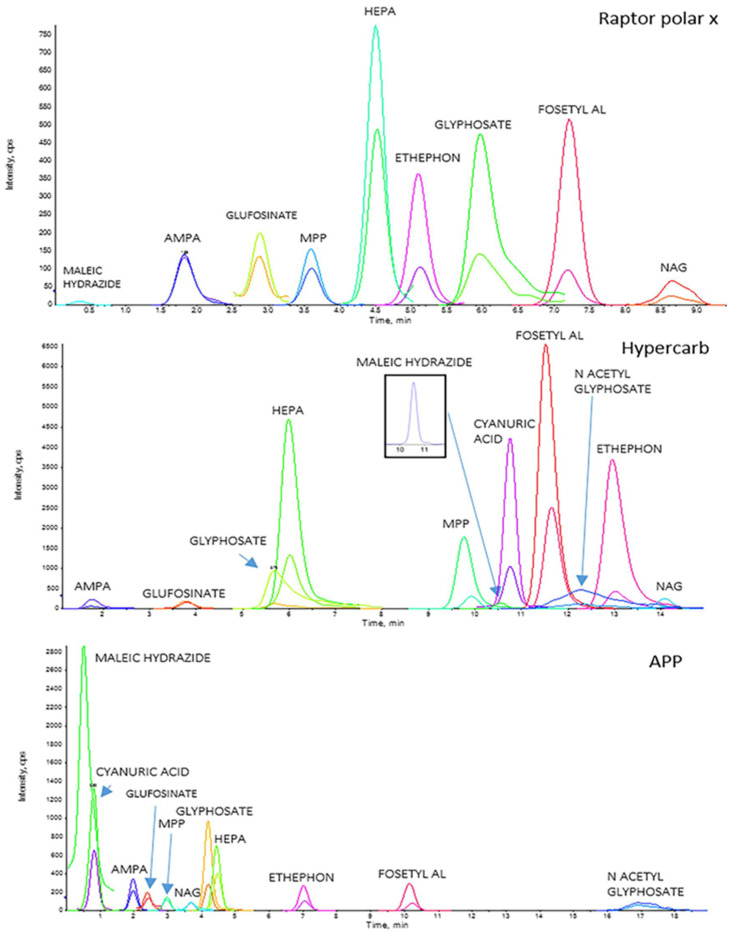
LC-HRMS chromatograms of chicken egg samples spiked with 0.005 mg/kg of AMPA, Etephon, Fosetyl Al, Glyphosate, HEPA, Maleic Hydrazide, N-Acetyl Glyphosate and Cyanuric Acid with 0.001 mg/kg for Glufosinate, MPP e N-Acetyl Glyphosate with the three columns of the present study: Raptor polar X (30 × 2.1 mm, 2.7 µm); Hypercarb™ (100 × 2.1 mm; 5 µm); Anionic Polar Pesticides (APP) (100 × 2.1 mm; 5 µm).

In Figure 11, it is possible to observe the traces obtained for the three columns for the eleven considered polar pesticides. The Hypercarb™ and the APP can separate all the defined analytes; the Raptor polar X instead did not give us the possibility to detect and quantify Cyanuric acid and N-Acetyl Glyphosate at a spike concentration of 0.005 mg/Kg.

The good performances obtained with the APP column have been confirmed by the European interlaboratory study [40] in which two participant groups used it, observing validation results compliant with the parameters required by the SANTE Document [17]. The results underscore the importance of selecting the appropriate chromatographic column based on the specific characteristics of the analytes and the matrix. The use of Hypercarb™ is particularly advantageous for glyphosate and its metabolites, same as Anionic polar pesticides, which offers a more comprehensive solution for this set of polar pesticides, showing great advantages in terms of peak shape and symmetry but some disadvantages concerning its stability. The Raptor Polar X™ column, despite its specialized design, showed limitations in terms of sensitivity for some analytes, indicating the need for further optimization in its application for polar pesticide analysis.

The study introduced an easy-to-apply novel total score-based methodology for evaluating the performance of chromatographic columns. This approach not only facilitated a comprehensive comparison in our research but also offered a robust framework for future column comparison projects. By adopting this methodology, researchers and analysts can efficiently determine the most suitable columns for their specific analytical needs, thereby enhancing the accuracy and reliability of pesticide detection in various matrices.

In the present study, the impact of several parameters on the chromatographic column selection has been evaluated in comparison with the results described in QuPPe, underscoring the importance of the development of new methodologies for an accurate and objective performance evaluation, as reported in Section 3.8.

The methodology detailed in this study can effortlessly be applied to a wide range of chromatographic systems and analytical methods. Furthermore, future research could expand these comparisons to include various animal-derived matrices, such as bovine muscle or fish, aiming to pinpoint the most effective chromatographic column for identifying polar pesticides in food. This expansion would not only enhance the versatility of the approach but also contribute to more accurate and reliable food safety assessments across different types of animal products.

Overall, our findings provide critical insights and a practical tool for improving the detection and quantification of polar pesticides in animal-origin products, contributing significantly to food safety and public health.

## Figures and Tables

**Table 1:** Characteristics of the three selected analytical columns.

Brand Name	Producer	Stationary Phase	Length (mm)	Internal Diameter (mm)	Particles (μm)
Hypercarb	Thermo	PGC ^1^	100	2.1	5
Raptor Polar	Restek	Hybrid Phase	30	2.1	2.7
Anionic Polar Pesticide	Waters	Diethylamine	100	2.1	5

^1^ Porous Graphitic Carbon.

**Table 5:** Analytes and acquisition method characteristics.

Analyte	Exp N°	Scan Type	Product of	Accumulation Time (Sec)	TOF MS Range (Da)	DP	CE
All	1	TOF MS	-	0.05	79–227	−50	−10
AMPA	2	Product Ion	110	0.1	50–115	−30	−33
Cyanuric Acid	3	Product Ion	128	0.05	30–130	−50	−24
	4	Product Ion	128.01	0.05	30–130	−50	−12
Etephon	5	Product Ion	143	0.05	50–150	−20	−9
	6	Product Ion	143.01	0.1	50–150	−20	−24
Fosetyl-Al	7	Product Ion	109	0.05	40–115	−40	−14
	8	Product Ion	109.01	0.05	40–115	−40	−10
Glufosinate	9	Product Ion	180	0.05	40–185	−50	−22
Glyphosate	10	Product Ion	168	0.05	40–175	−45	−15
	11	Product Ion	168.01	0.05	40–175	−45	−24
HEPA	12	Product Ion	125	0.05	40–130	−50	−26
	13	Product Ion	125.01	0.05	40–130	−50	−74
Maleic Hydrazide	14	Product Ion	111	0.1	70–115	−70	−20
MPP	15	Product Ion	151	0.05	50–160	−30	−15
	16	Product Ion	151.01	0.05	50–160	−30	−48
N-Acetyl Glufosinate	17	Product Ion	222	0.05	50–230	−50	−27
	18	Product Ion	222.01	0.05	50–230	−50	−65
N-Acetyl Glyphosate	19	Product Ion	210	0.05	130–220	−50	−20
	20	Product Ion	210.01	0.05	50–220	−50	−40

**Table 6:** Points were awarded to the analytical columns for the conditioning criterion.

Column		Hypercarb	Raptor Polar X	APP
Conditioning/Passivation	Yes	X	X	
	No			X
Points		0	0	5

For the Conditioning/Passivation criterion, APP represents the best choice.

**Table 7:** Points were awarded to the analytical columns for peak Shape and Symmetry. For AMPA and Glyphosate, the points are doubled.

Column	Hypercarb		Raptor Polar X		APP	
Analyte	Peak	Score	Peak	Score	Peak	Score
AMPA	UAAP	6	UAAP	6	UASP	8
Glyphosate	DAAP	2	DAAP	2	UASP	8
Etephon	UASP	4	UASP	4	UASP	4
Glufosinate Ammonium	UASP	4	UASP	4	UASP	4
MPP	UASP	4	UASP	4	UASP	4
N-Acetyl Glufosinate	UASP	4	UASP	4	UASP	4
N-Acetyl Glyphosate ^1^	DAAP	1	N.A.	0	DASP	2
HEPA	UASP	4	UASP	4	UASP	4
Cyanuric Acid ^1^	UASP	4	N.A.	0	UASP	4
Maleic Hydrazide	UASP	4	UASP	4	UASP	4
Fosetyl Al	UASP	4	UASP	4	UASP	4
Total Points	41		36		50	

^1^ Analytes not detected with Raptor Polar X.

**Table 8:** RSD for Area and Retention time for the three selected analytes.

Analyte	RSD	Hypercarb		Raptor Polar X		APP	
		Value	Points	Value	Points	Value	Points
Glyphosate	Area	8.1	4	29.6	2	31.5	0
	RT	2.1	4	2.7	4	4.3	4
Glufosinate Ammonium	Area	14.2	3	17.2	3	13.8	3
	RT	1.2	4	2.1	4	1.6	4
N-Acetyl Glufosinate	Area	20.9	2	17.2	3	29.6	2
	RT	0.8	4	0.8	4	1.6	4
Total Points		21		20		17	

**Table 9:** Points were awarded for the Sensitivity test.

Column	Hypercarb		Raptor Polar X		APP	
Analyte	LOD Value (ng/g)	Score	LOD Value (ng/g)	Score	LOD Value (ng/g)	Score
AMPA	1.38	3	0.82	5	0.99	4
Glyphosate	0.95	3	0.53	5	0.80	4
Etephon	0.27	5	0.70	4	1.04	3
Glufosinate Ammonium	0.12	5	0.81	4	1.61	3
MPP	0.23	4	0.80	3	0.17	5
N-Acetyl Glufosinate	0.33	5	0.90	3	0.51	4
N-Acetyl Glyphosate ^1^	0.90	4	N.A.	0	1.59	5
HEPA	0.25	4	0.50	3	0.16	5
Cyanuric Acid ^1^	0.79	4	N.A.	0	1.21	3
Maleic Hydrazide ^1^	0.64	4	N.A.	0	0.83	3
Fosetyl Al	0.03	5	0.39	4	1.77	3
Total Points	46		31		42	

^1^ Analytes not detected with Raptor Polar X.

**Table 10:** Retention factor (k) test results for Hypercarb, Raptor Polar X, and APP.

Column	Hypercarb			Raptor Polar X			APP		
Analyte	RT	k	Score	RT	k	Score	RT	k	Score
AMPA	1.76	0.60	0	1.85	7.45	1	1.97	3.47	1
Glyphosate	5.33	3.84	1	6.52	21.95	0	4.18	8.47	1
Etephon	13.02	10.81	0	5.57	24.47	0	7.04	14.97	0
Glufosinate Ammonium	3.71	2.37	1	2.94	12.48	0	2.40	4.44	1
MPP	10.35	8.39	1	3.95	17.06	0	2.96	5.72	1
N-Acetyl Glufosinate	14.64	12.28	0	10.18	45.54	0	3.67	7.34	1
N-Acetyl Glyphosate ^1^	17.86	16.20	0	--	--	--	17.08	37.74	0
HEPA	5.66	4.14	1	4.83	21.09	0	4.44	9.08	1
Cyanuric Acid ^1^	11.25	9.21	1	--	--	--	0.81	0.85	0
Maleic Hydrazide	11.04	9.02	1	0.35	0.41	0	0.93	1.11	1
Fosetyl Al	11.54	9.47	1	7.76	27.93	0	10.19	22.12	0
Total Points	7			1			7		

^1^ Analytes not detected with Raptor Polar X.

**Table 11:** Summarized results for RSD, RT, and Symmetry of the analyte’s peaks after 500 injections.

Analyte	RSD Value	Hypercarb		Raptor Polar X		APP	
		Value	Score	Value	Score	Value	Score
AMPA	RT	1.9	2	1.5	3	0.3	4
	Symmetry	26.5	2	23.5	3	9.9	4
Glyphosate	RT	5.8	3	14.7	2	1.7	4
	Symmetry	38.8	2	27.9	3	13.0	4
Etephon	RT	3.2	3	13.5	2	1.0	4
	Symmetry	16.3	3	20.1	2	13.5	4
Glufosinate Ammonium	RT	2.4	4	6.1	2	3.8	3
	Symmetry	23.7	4	24.3	3	40.4	2
MPP	RT	4.8	3	14.4	2	2.1	4
	Symmetry	26.6	2	14.7	3	11.3	4
N-Acetyl Glufosinate	RT	4.8	3	24.5	2	1.8	4
	Symmetry	33	3	43.5	2	18.5	4
N-Acetyl Glyphosate ^1^	RT	3.78	3	--	--	0.9	4
	Symmetry	13.8	3	--	--	62.4	4
HEPA	RT	5.2	3	11.2	2	1.5	4
	Symmetry	18.2	2	13.3	3	8.6	4
Cyanuric Acid ^1^	RT	3.3	3	--	--	0.9	4
	Symmetry	9.6	4	--	--	11.2	3
Maleic Hydrazide	RT	3.3	3	4.4	2	1.8	4
	Symmetry	13.2	3	25.5	2	10.3	4
Fosetyl Al	RT	2.6	3	11.7	2	0.7	4
	Symmetry	12.2	3	11.6	4	15.9	2
Total Points		64		44		82	

^1^ Analytes not detected with Raptor Polar X.

**Table 12:** Points for any target analyte provided by the validation method are separated by the columns.

Analyte	Hypercarb		Raptor Polar X		APP	
	Detection	Score	Detection	Score	Detection	Score
AMPA	X	1	X	1	X	1
Glyphosate	X	1	X	1	X	1
Etephon	X	1	X	1	X	1
Glufosinate	X	1	X	1	X	1
MPP	X	1	X	1	X	1
N-Acetyl Glufosinate	X	1	X	1	X	1
N-Acetyl Glyphosate	X	1	--	0	X	1
HEPA	X	1	X	1	X	1
Cyanuric Acid	X	1	--	0	X	1
Maleic Hydrazide	X	1	X	1	X	1
Fosetyl Al	X	1	X	1	X	1
Total Points	11		9		11	

**Table 13:** Points for any extra molecule are not considered in the validation method but are separated by the column.

Analyte	Hypercarb		Raptor Polar X		APP	
	Detection	Score	Detection	Score	Detection	Score
Bialaphos	--	0	X	0.5	X	0.5
Bromate	X	0.5	--	0	--	0
Bromide	X	0.5	--	0	X	0.5
Chlorate	X	0.5	--	0	X	0.5
Desmethyl-Dismethoate	--	0	X	0.5	--	0
Perchlorate	X	0.5	--	0	X	0.5
Phosphonic Acid	X	0.5	--	0	X	0.5
Thiocyanate	X	0.5	--	0	--	0
Trifluoroacetic Acid	--	0	--	0	X	0.5
Total Points	3		1		3	

**Table 14:** Total Score for the three columns considered in the present study.

Total Points		
Hypercarb	Raptor Polar X	Anionic Polar Pesticide
193	142	207

## Data Availability

The original contributions presented in the study are included in the article, further inquiries can be directed to the corresponding author/s.
